# Is Excess Calcium Harmful to Health?

**DOI:** 10.3390/nu2050505

**Published:** 2010-05-17

**Authors:** Robin M. Daly, Peter R. Ebeling

**Affiliations:** Department of Medicine (RMH/WH), The University of Melbourne, Western Hospital, Melbourne 3011, Australia; Email: peterre@unimelb.edu.au

**Keywords:** calcium supplementation, cardiovascular disease, mortality, elderly

## Abstract

Most current guidelines recommend that older adults and the elderly strive for a total calcium intake (diet and supplements) of 1,000 to 1,300 mg/day to prevent osteoporosis and fractures. Traditionally, calcium supplements have been considered safe, effective and well tolerated, but their safety has recently been questioned due to potential adverse effects on vascular disease which may increase mortality. For example, the findings from a meta-analysis of randomized controlled trials (currently published in abstract form only) revealed that the use of calcium supplements was associated with an ~30% increased risk of myocardial infarction. If high levels of calcium are harmful to health, this may alter current public health recommendations with regard to the use of calcium supplements for preventing osteoporosis. In this review, we provide an overview of the latest information from human observational and prospective studies, randomized controlled trials and meta-analyses related to the effects of calcium supplementation on vascular disease and related risk factors, including blood pressure, lipid and lipoprotein levels and vascular calcification.

## 1. Introduction

Maintaining an adequate calcium intake through dietary sources or supplements is widely regarded as a safe and effective strategy for the prevention and treatment of osteoporosis. In both postmenopausal women and older men calcium supplementation or increased dietary calcium through fortified milk has been shown to slow or prevent bone loss at common fracture sites such as the hip and spine [1,2,3,4,5]. It is likely that these positive effects on bone density translate into anti-fracture efficacy, as the findings from a recent meta-analysis of randomized controlled trials (RCTs) in adults aged 50 years and older reported that calcium supplementation or combined calcium and vitamin D supplementation reduced the relative risk of fractures by 12% [6]. At present, most dietary guidelines recommend that older adults and the elderly aim for a habitual total calcium intake (diet and supplements) of 1,000 to 1,300 mg per day. These requirements are based largely on data from calcium balance studies and the determination of the ‘maximal calcium retention’ or the threshold value at which balance does not further improve as intake increases [7]. However, recent data based on calcium intakes around predicted zero balance suggests that calcium requirements should be in the range of 741 to 1035 mg/d for healthy adults, regardless of age or sex [8]. While it is beyond the scope of this review to debate the specific merits associated with the determination of calcium requirements for adults, it is important to highlight that many elderly people do not obtain sufficient calcium through dietary sources alone. As a result, calcium supplements are widely recommended and have traditionally been considered a safe alternative to meeting calcium requirements. The most common side effects relate to bloating, constipation and more uncommonly, a slightly increased risk of kidney stones [9].

Over the past two decades there has been a growing body of evidence to suggest that the use of calcium [13,14,15,16,17,18,19] or calcium-vitamin D supplements [10,11,12] may lead to a number of non-skeletal health benefits, including beneficial effects on cardiovascular disease (CVD) and related risk factors, type 2 diabetes, certain cancers and even mortality. For instance, several clinical trials and meta-analyses have reported that calcium supplementation may improve serum lipids concentrations [15,16,17] and blood pressure [18,19]. Based on these findings, one might expect that increased calcium would have protective effects on various cardiovascular endpoints, such myocardial infarction (MI), coronary heart disease (CHD) or stroke. However, there is emerging data from various studies (observational, an RCT and a meta-analysis) that long-term use of calcium supplements in healthy older adults, particularly women, may not be safe because it could have an adverse effect on cardiovascular outcomes [20,21,22]. Because ageing is also associated with an increased incidence of CVD, even a slight increase in adverse vascular events would be clinically important because it might counterbalance any beneficial effects of calcium supplements in preventing osteoporosis and fractures.

This review provides an overview of the latest evidence relating to the effects of calcium supplementation on vascular disease and related risk factors and associated mortality in the elderly. Several reviews have recently evaluated the impact of dairy foods on cardiovascular disease risk [23,24], and thus we will not focus on their potential effects. However, the general consensus from these reviews is that dairy food consumption is not associated with a higher risk of CVD. 

## 2. Calcium Supplementation, Cardiovascular Disease and Mortality

There have been a number of large observational epidemiological and prospective studies which have investigated the association between calcium intake and calcium supplement use, and cardiovascular disease risk and related mortality in older women and men. Typically, the findings from these studies indicate either a neutral effect [25,26,27] or a modest protective effect of a high total calcium intake against vascular disease and even mortality [10,14,28]. With regard to the specific effects of calcium supplements however, most studies to date have reported a non-significant effect. For example, in a prospective study of 34,486 postmenopausal Iowa women aged 55–69 years followed for 8 years, the relative risk of ischemic heart disease (IHD) mortality was 0.67 (95% CI, 0.47–0.94) for women in the highest compared to lowest quartile for total calcium intake (diet + supplements) [14]. In contrast, for women (n = 56) taking >500 mg/d of calcium supplements there was no significant risk reduction [multivariate relative risk (RR) 0.88 (95% CI, 0.64, 1.23)] [14]. Similar non-significant findings with regard to calcium supplement use were reported in a 12 year prospective study in 39,800 men aged 40–75 years enrolled in the Health Professionals Follow Study [25]. In this study, the multivariate RR for developing IHD in the highest quintile of calcium supplement use (median 1000 mg/d) compared with non-users was 0.87 (95% CI, 0.64, 1.19); the RR for nonfatal MI and IHD were 1.02 (95% CI, 0.71, 1.46) and 0.61 (95% CI, 0.34, 1.10), respectively [25]. Two other large studies also reported that the RR of stroke was no different in men or women who received 400 mg/d or more of supplemental calcium compared with non-users [26,27]. Data from a small study of acute hip fracture patients showed that the use of prescribed calcium and vitamin D supplements post-fracture was associated with a 36% and 43% reduction in death in women and men, respectively, after 3 years [28]. Contrary to all these findings, a recent prospective study in 10,555 Finnish women aged 52-62 years who were free of coronary heart disease (CHD) at baseline and followed for a mean of 6.55 years, reported that women using calcium supplements (or calcium plus vitamin D) had a 24% increased risk of CHD compared to non-users [1.24 (95% CI, 1.02, 1.52)] [21]. It is somewhat difficult to explain these findings given that the average age of the women was 57 years, and those taking supplements had a lower BMI, were more often non-smokers or former smokers and had less diabetes and hypertension mortality than non-users. 

**Figure 1:**
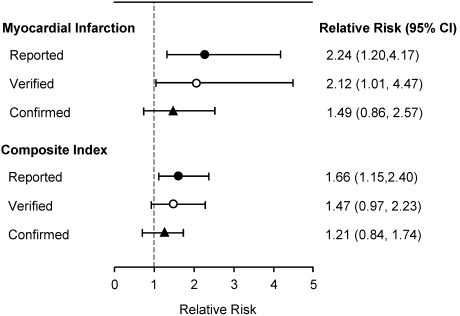
Summary of the relative risk for myocardial infarction (MI) and the composite endpoint of MI, stroke or sudden death based on self report, verified (reported events verified by a physician) and confirm events (includes verified events and events obtained from the New Zealand national database on hospital admissions) in healthy postmenopausal women in the calcium supplementation compared to placebo group involved in the Auckland Calcium Study [20].

Although there are no RCTs that have specifically evaluated the effect of calcium supplement use on CVD events or related mortality, a number of studies have pre-specified or performed secondary analyses to assess the effect of calcium supplementation on various cardiovascular endpoints, including MI, stroke, CHD, sudden death or their combination (Table 1). Of these, the findings from a recent preplanned secondary analysis of a 5-year RCT of 1471 healthy, elderly postmenopausal women who received calcium citrate (1,000 mg/d) or placebo has received the most attention because it has raised concerns about the cardiac safety for supplemental calcium [20]. However, an accompanying editorial highlighted that data from this study were not totally consistent, and thus must be interpreted cautiously [29]. In the analysis based on self-report and then adjudication by physicians (verified), those receiving calcium supplementation had a increased risk of MI (RR self report 2.24, p < 0.001; RR verified 2.12, p < 0.05) and the composite endpoint of MI, stroke or sudden death (RR self report 1.66, p < 0.01; RR verified 1.47, p = 0.08) [30] (Figure 1). When the authors then searched the New Zealand hospital database of hospital admissions to identify additional vascular events that might have been missed, no statistically significant differences were detected between the calcium and placebo groups for either MI [RR 1.49 (95% CI, 0.86. 2.57)] or the composite endpoint [RR 1.21 (95% CI, 0.84, 1.74)] [30] (Figure 1). However, rate ratio analysis revealed a trend for an increased risk of MI (1.67, p = 0.058) and the composite endpoint (1.43, p = 0.043). These findings do not provide conclusive evidence, although surprisingly the authors maintain that ‘..*there are reasonable grounds for doubting the safety of calcium supplements*..’ and suggest ‘.. *there should be a reappraisal of their role in the management of osteoporosis*..’ [31]. This view is not universally shared. Other investigators have raised concerns about various aspects of this study, including the relatively small number of vascular events and the wide confidence intervals, the borderline significance of many of the findings, and the failure to consider other important confounders, including serum 25-hydroxyvitamin D [32,33]. It is therefore critical that the results from other similar calcium supplementation studies be considered when evaluating the clinical and public health significance of these findings. 

A summary of the key findings from the various RCTs that have evaluated the effects of calcium supplementation or combined calcium-vitamin D supplementation on cardiovascular related endpoints is presented in Table 1. In all these previous RCTs (with the exception of the Auckland study) there were no statistically significant adverse (nor beneficial) effects of calcium supplementation on CVD risk. For example, in an Australian study of 1460 postmenopausal women aged ≥70 years who were randomized to take calcium carbonate (1,200 mg/d) or placebo for 5 years, Prince *et al.* [3] reported that incident IHD was no different between the supplemented and placebo group [HR 1.12 (95% CI, 0.77,1.64)]. Similarly, secondary analysis of a 4-year RCT in 1179 postmenopausal women aged >55 years in the United States found no excess occurrence of MI or other vascular events in the calcium treated (1,400–1,500 mg/d calcium citrate or carbonate) compared to placebo group [34]. However, the participants in this study were ~10 years younger than the Auckland women. Despite this, it is intriguing that both these trials were similar to the Auckland study in terms of their design, study duration, number of participants and baseline calcium intakes (~850–1,000 mg/d), but failed to observe any significant adverse effects. In addition, the dose of calcium was less in the Auckland study (1,000 mg/d *versus* 1,200–1,500 mg/d). However, one possible explanation for the contrasting results may relate to the type of calcium supplements used. Women in the Auckland trial consumed calcium citrate, whereas calcium carbonate was used in the Australian study and carbonate or citrate in the US trial. Calcium citrate has been reported to have superior bioavailability compared with calcium carbonate [35], and leads to a greater acute rise in ionized calcium concentration [36]. These findings, together with the fact that calcium carbonate must be taken with meals, may be one explanation for the contrasting results. Differences in serum 25-hydroxyvitamin D [25OHD] levels may also be a contributing factor given that low vitamin D has been associated with an adverse cardiovascular risk profile and increased risk of CV events [37,38]. Serum 25OHD levels were not reported in the Auckland study, but in the Australian and US trials levels ranged from a mean of 67 to 72 nmol/L.

When evaluating the potential adverse effects of calcium supplement use on vascular events and related mortality, it is also worth considering data from two of the largest trials [RECORD study and the Women’s Health Initiative (WHI)] that were designed to examine the effects of calcium carbonate and/or vitamin D on fracture incidence [39,41]. In both these studies, no significant overall effect of supplement use on cardiovascular event rates or mortality was observed [10,39,41]. The RECORD Study did not report vascular events, but there were no differences in death rates between calcium supplement users and non-users (17.7% *vs.* 16.2%) [39]. In the WHI trial involving over 36,000 postmenopausal women, the HR was 1.04 (95% CI, 0.92–1.18) for MI or CHD death [41] and 0.91 (95% CI, 0.83, 1.01) for total mortality [10]. There was a trend for an increase in a composite end-point of MI, death from CHD, coronary artery bypass graft or percutaneous coronary intervention [HR 1.08 (95%CI 0.99–1.18)] [41]. A major limitation of this study is that over 50% of the women were using non-study calcium supplements. Interestingly, when the analysis was restricted to women not self administering calcium, there was a 17% significant increased risk of MI or coronary revascularization [HR 1.17 (95% CI, 1.01, 1.36)] [22]. However, in a subsequent study the authors reported that in women younger than 70 years of age, there was a trend toward a reduction in the risk of total, cardiovascular and cancer mortality in those receiving calcium plus vitamin D supplementation [10]. When interpreting these findings and those from the RECORD study, it is important to consider the use of vitamin D given the evidence that supplemental vitamin D may reduce cardiovascular disease risk and all-cause mortality [43]. Other potential important confounding factors must also be considered when evaluating the results of the WHI study, including non-compliance and the use of HRT.

**Table 1:** Summary of the results from randomised controlled trials that evaluated the effects of calcium supplementation or combined calcium-vitamin D supplementation on cardiovascular related endpoints.

Reference	Participants	Age	Intervention	Mean Dietary Ca	Mean Total Ca ^†^	Duration	Primary Study Endpoint	Adverse event recording method	Key Findings
Baron *et al.* [13].	930 men and women (recent history of colorectal adenomas)	Mean 61 yrs	Calcium carbonate, 1200 mg/d (n = 464); placebo (n = 466).	Ca, 889 mg/d; placebo, 865 mg/d.	Ca, ~2089 mg/d	4 yrs	Recurrent colorectal adenoma incidence	Hospitalized events	The number of participants hospitalized for cardiac events was no different between the Ca supplemented (n = 50, 11%) and placebo group (n = 46, 10%). Death rates were also similar between the groups (5% each in the calcium and placebo group).
Grant *et al.* [39].	5292 men and women	≥70 yrs	Calcium carbonate, 1000 mg/d + vitamin D_3_, 800 IU/d (n = 1306); calcium carbonate, 1000 mg/d (n = 1311); vitamin D_3_, 1000 IU/d (n = 1343); placebo (n = 1332).	Not reported	-	2 yrs to 62 months	Low energy trauma fracture incidence	Self reported adverse events, death.	No differences in death rates between the four groups: Ca+Vit D (16.9%); Vit D_3_ (16.2%); Ca (18.5%); placebo (16.3%), or the combined Ca *vs.* non-Ca groups (17.7% *vs.* 16.2%).
Brazier *et al.* [40].	192 women	>65 yrs	Calcium carbonate, 1000 mg/d + vitamin D, 800 IU/d (n = 95); placebo (n = 96).	Ca + Vit D, 752 mg/d; placebo, 721 mg/d.	Ca + Vit D, ~1752 mg/d	1 yr	Bone density, biochemical markers	Self reported and observed adverse events.	No difference in adverse cardiovascular events or death between the participants in the Ca+Vit D (n = 6) and placebo group (n = 5).
Prince *et al.* [3].	1460 women	>70 yrs	Calcium carbonate, 1200 mg/d (n = 730); placebo (n = 730).	Ca, 915 mg/d; placebo, 897 mg/d.	Ca, ~2115 mg/d	5 yrs	Clinical incident fractures, vertebral deformity and adverse events	Self reported adverse events recorded in a dairy every 4 months.	Incident ischemic heart disease was no different between the Ca (56 participants, 7.7%) and placebo group (51 participants, 7.0%); HR, 1.12 (95% CI 0.77-1.64).
Hsia *et al.* [41].	36,282 postmenopausal women	50 to 79 yrs	Calcium carbonate, 1000 mg/d + vitamin D_3_, 400 IU/d (n = 18,176); placebo (n = 18,106).	Diet and supplements; Ca, 1148 mg/d; placebo, 1154 mg/d.	Ca, ~2148 mg/d	7 yrs	Fracture incidence	Medical review of self reported events; adjudicated by centrally trained physicians	No differences between the Ca+Vit D and placebo group for various CV events (RR): MI/CHD, 1.04 (95% CI 0.92, 1.18); MI, 1.05 (95% CI, 0.91, 1.20); CHD death, 1.01 (95% CI, 0.79, 1.29); stroke, 0.95 (95% CI, 0.83, 1.10); transient ischemic attack, 1.16 (95% CI, 0.95, 1.42).
Bolland *et al.* [20].	1471 postmenopausal women)	Mean 74 yrs	Calcium citrate, 1000 mg/d (n = 732); placebo (n = 739).	Ca, 861 mg/d; placebo, 853 mg/d.	Ca, ~1861 mg/d	5 yrs	Bone density and fracture incidence	Self reported adverse events, review of hospital records and death certificates; search of national database of hospital admissions for CV events.	For verified vascular events, the RR in the Ca vs placebo groups were as follows: MI 1.49 (95% CI, 0.86, 2.57); stroke 1.37 (95% CI, 0.83, 2.28); sudden death 0.51 (95% CI, 0.13, 2.01); MI, stroke or sudden death 1.21 (95% CI, 0.84, 1.74).
Reid *et al.* [4].	323 men	≥40 yrs	Calcium citrate, 1200 mg/d (n = 108); calcium citrate, 600 mg/d (n = 108); placebo (n = 107).	Ca (1200 mg/d), 930 mg/d; Ca (600 mg/d), 870 mg/d; placebo, 800 mg/d.	Ca (1200 mg/d), ~2130 mg/d; Ca (600 mg/d), ~1470 mg/d	2 yrs	Bone density	Self reported adverse events.	No difference between Ca groups and placebo for composite vascular events (angina, MI, sudden death, coronary revascularization) (p = 0.24); Ca 1200 mg/d (n = 3); Ca 600 mg/d (n = 2), placebo (n = 0).
Lappe *et al.* [34].	1179 postmenopausal women	>55 yrs	Calcium citrate or carbonate (1400-1500 mg/d) + vitamin D (1000 IU/d) (n = 446); calcium (as above) + vitamin D placebo (n = 445); double placebo (n = 288).	Diet and supplements, mean Ca, 1072 mg/d.	Ca, ~2400 mg/d	4 yrs	Fracture incidence	Self reported adverse events at 6 monthly intervals, which were then verified by the participants physician.	Vascular event rate in the two Ca treatment groups combined was no different from the placebo group (4.76 events/1000 person year *vs.* 6.94 events/1000 person year).
Zhu *et al.* [42].	120 women	70-80 yrs	Calcium carbonate, 1200 mg/d + vitamin D2, 1000 IU/d (n = 39); Calcium carbonate, 1200 mg/d + placebo vitamin D2 (n = 40), placebo calcium and vitamin D (n = 41)	Ca + vit D, 927 mg/d; Ca 1054 mg/d; placebo, 1046 mg/d.	Ca + Vit D, ~2127 mg/d; Ca, ~2254 mg/d	5 yrs	Bone density and biochemical markers	Self reported adverse events	There were no significant differences among the three groups in the rate of incident vascular disease (data not reported).

† Mean total calcium (Ca) intake represents the estimated intake based on the mean baseline dietary calcium intake combined with the dose of supplemental calcium used in the intervention.

In light of the emerging concern that a high supplemental calcium intake may be associated with an increased risk for cardiovascular events, two recent studies have reviewed the available calcium supplementation RCTs that included cardiovascular endpoints [22,44]. Wang *et al.* [44] conducted a systematic review of prospective studies and RCTs that examined calcium supplementation, vitamin D supplementation or both on subsequent cardiovascular events. When the data from the RCTs were combined, the RR for CVD was 1.14 (95% CI, 0.92, 1.41) for calcium supplements *versus* placebo, and 1.04 (95% CI, 0.92, 1.18) for combined calcium plus vitamin D *versus* double placebo. There was also no significant effect of vitamin D supplementation on CVD [RR 0.90 (95% CI, 0.77, 1.05)] [44]. Based on these findings, the authors concluded that calcium supplements seem to have minimal cardiovascular effects, but a limitation of this study is that only a small number of trials were included in the meta-analysis. A more recent and comprehensive meta-analysis included a larger number of trials (n = 15), with patient-level data available for five studies (n = 8151), and trial-level data for 11 studies (n = 11,921) with a mean follow-up of 4 years [22]. The inclusion criteria for this study were placebo controlled trials >12 months in duration with a calcium supplement dose >500 mg/d and at least 100 participants [22]. For participants allocated to calcium supplements, the HRs for MI were 1.31 (95% CI, 1.02, 1.67) and 1.27 (95% CI, 1.01, 1.59) for the patient- and trial- level data, respectively. This indicates that the use of calcium supplements was associated with an ~30% increase in cardiovascular disease risk. However, since these results are currently only available in abstract form, it is difficult to provide a more comprehensive evaluation of the methodology and study findings. 

## 3. Calcium Supplementation, Lipids and Blood Pressure

If the use of calcium supplements is associated with an increased risk of cardiovascular disease, one might expect that other common cardiovascular risk factors, such as cholesterol, lipids and blood pressure, would also be adversely affected. Contrary to this hypothesis, the findings from a number of clinical trials have shown that calcium supplementation was associated with improvements in serum lipids concentrations, particularly in women [15,16,17]. This effect is reported to be due to the binding of calcium to fatty acids and bile acids in the intestines, which leads to a reduction in fat absorption [16,45]. It has also been proposed that a reduction in parathyroid hormone (PTH) and/or 1,25-dihydroxyvitamin D following calcium supplementation may lead to a decrease in intracellular calcium which reduces calcium flux into adipocytes [46]. This may then inhibit lipogenesis and promote lipolysis [46], leading to lower adiposity and thus favorable effects of lipid and lipoprotein levels. This proposed effect of calcium on adipocyte lipid metabolism, along with the reported pressor effects of calcium on vascular smooth muscle cells [47], may also explain the findings that calcium supplementation can lower the risk of hypertension. Results of several meta-analyses of RCTs indicate that calcium supplementation can lead to small but significant reductions in both systolic and diastolic blood pressure [18,19], with the greatest blood-pressuring lowering effects observed in those with the lowest baseline calcium intakes ( < 600–800 mg/d) before supplementation [19,48] and/or those who are hypertensive [49]. Based on these findings, it would appear that any potential adverse effects of calcium on cardiovascular endpoints are unlikely to be secondary to changes in either lipids or blood pressure. 

## 4. Calcium Supplementation and Vascular Calcification

Vascular calcification is a marker of sub-clinical atherosclerotic disease, and an independent predictor of subsequent vascular morbidity and mortality in men and women, including CHD, CVD and mortality [50]. In patients with kidney disease, there is evidence from clinical trials showing that the use of calcium supplements is associated with increased vascular calcification [51]. For example, the results from a 12 month trial in 200 hemodialysis patients comparing calcium acetate or carbonate with a non-calcium based binder (sevelamer) showed that calcium supplementation lead to a more rapid increase in both coronary and aortic calcification [52]. In the Auckland Calcium Study, the authors speculated that the upward trends in cardiovascular event rates could be related to the age of the participants and their associated reduction in renal function [20]. The mean glomerular filtration rate (GRF) of the women in this study was 61 (SD 11) mL/min [31], which indicates that a portion of women had impaired renal function. In 90 pre-dialysis patients with a mean GRF of ~30 mL/min, supplementation with calcium carbonate for an average of 2 years resulted in a significant increase in coronary artery calcification (CAC), but these changes in total calcium scores (TCS) were not significantly different from controls (178 *vs.* 205) [53]. Therefore, it is questionable whether impaired renal function is a likely reason for the increased vascular risk in the postmenopausal women receiving calcium in the Auckland trial. 

In apparently healthy older men and women, there are few studies which have investigated the effects of increased calcium on the progression of vascular calcification. Bhakta *et al.* [54] conducted a retrospective analysis in a subgroup of participants (n = 257) enrolled in the prospective Epidemiology of Coronary Artery Calcification study in Minnesota who were aged >60 years and had complete 4-year follow-up on aortic value calcification (AVC) as assessed by electron beam computed tomography (EBCT). In women, the progression of AVC or CAC over a mean of 3.7 years was no different between those who reported using calcium supplements (n = 25) and those who did not (n = 114); no men in the study reported using calcium supplements. Similarly, the findings from a 2-year RCT involving 163 healthy men aged 57 ± 10 years who had taken 1200 mg/d of supplemental calcium or placebo revealed that supplementation was not associated with an increase in coronary calcification assessed by computed tomography (CT) [55]. In contrast, in a retrospective analysis of a 2-year RCT, we reported that supplementing the diet with calcium and vitamin D_3_ fortified milk (1,000 mg/d and 800 IU/d) may accelerate abdominal aortic calcification (AAC) measured by CT in older men, but only in those who presented with AAC prior to the commencement of the study [56]. In this subgroup of men, who had a mean dietary calcium intake at study entry of ~900 mg/d, the mean AAC scores increased significantly in the milk relative to control group [mean change (95%CI); 24 (7, 42); controls 6 (-5, 18), P < 0.05], independent of age, BMI, anti-hypertensive or lipid-lowering medication use, smoking, exercise or blood pressure [56]. It is difficult to explain these results given that there were no differences between the fortified milk and control groups for changes in weight, fat mass, blood pressure, lipids, serum calcium, GFR, fat or saturated fat intake. In addition, baseline serum 25OHD levels were ~75 nmol/L, and increased further following supplementation which might be expected to have a protective effect.

The development of vascular calcification is a complex process that is not only dependent on the physio-chemical effects of calcium and phosphate, but also on factors that can regulate the differentiation of smooth muscle cells to osteoblast-like cells; similar to those in osteogenesis. Potential mediators include both inhibitors [pyrophosphate; osteoprotegerin (OPG); osteopontin, matrix Gla protein (MGP), fetuin A, parathyroid hormone-related protein (PTHrP); fibroblast growth factor 23 (FGF 23)] and activators [phosphorus, calcium, alkaline phosphatase; bone morphogenetic protein 2, 1,25(OH)_2_D; osteocalcin; osteonectin; oxidized low density lipoprotein (LDL), advanced glycation end products (AGEs)] that regulate this process [51]. Previous research has shown that serum OPG, which inhibits osteoclast differentiation and activity and stimulates osteoclast apoptosis, is related to cardiovascular events [57], and the presence and/or severity of aortic calcification in patients undergoing haemodialysis [58] or those with peripheral vascular disease [59]. However, in 80 healthy older women from the Auckland Calcium Study, no differences were observed for the changes in serum OPG and MGP in women treated with calcium or placebo for 5 years [31]. 

## 5. Calcium Supplementation and Cerebrovascular Disease Risk

Some of the early work examining the relationship between calcium and cerebrovascular disease suggests that there may be a protective effect of calcium. For example, in an ecologic study of elderly people from the southwest of France, a high level of calcium in drinking water was associated with a lower risk of noncerebrovascular (10%) and cerebrovascular (14%) related death [60]. Calcification of vascular smooth muscle has also been associated with brain lesions, which are a marker of cerebrovascular disease. In the elderly, there is some evidence that a high calcium intake is associated with brain lesions. Payne *et al.* [61] examined the relationship between dietary calcium and vitamin D intakes and brain lesions, measured by MRI scans, in 232 elderly men and women (95 with current or prior depression, 137 without depression). They found that both calcium and vitamin D were positively associated with a higher total volume of brain lesions, even after controlling for potential confounders, including age, hypertension, diabetes, heart disease, kilocalories, group (depression or no depression), and lesion load (high or low). Furthermore, the authors’ earlier work found that individuals consuming high-fat dairy products (1.8 serves per day) had 1.5 times greater brain lesion volume than those consuming 0.3 serves per day [62]. Although cause-and-effect cannot be established from these cross-sectional studies, the apparent link between calcium and brain lesions warrants further research given that brain lesions have also been linked to an increased risk of cognitive impairment, dementia, and depression.

## 6. Calcium Supplementation and Kidney Stones

Kidney stones have been linked to a high calcium intake, but this appears to depend on the source of calcium. Several prospective studies reported that a diet high in calcium is associated with a reduced risk of kidney stones, possibly by reducing gut absorption of oxalate which is one of the main components of kidney stones [63,64]. In contrast, the use of calcium supplements has been associated with an increased risk, although these finding are not consistent. For instance, data from the 7-year Women’s Health Initiative (WHI) trial revealed that the risk of kidney stones (renal calculi) was increased by 17% in those receiving calcium and vitamin D supplements [HR 1.17 (95% CI, 1.02, 1.34) [9]. In contrast, a systematic review of calcium supplementation trials in postmenopausal women revealed that most studies show no increase in stone risk with a high calcium intake (diet or supplements), and in fact several trials reported an inverse association between calcium intake and stone risk [65].

## 7. Serum Calcium and Cardiovascular Disease Risk

It has been suggested that the increased risk of vascular disease with calcium supplementation may be related to serum calcium concentrations [31]. There is indirect evidence to support this hypothesis. A number of observational and prospective studies in men and women have reported that serum calcium levels were related to the risk of MI [66,67] and even death [67]. Data from 40,538 hemodialysis patients showed serum phosphorus concentrations >5.0 mg/dl were associated with an increased relative risk of death (RR, 1.07, 1.25, 1.43, 1.67, and 2.02 for serum phosphorus at 5.0 to 6.0, 6.0 to 7.0, 7.0 to 8.0, 8.0 to 9.0, and ≥9.0 mg/dL) [68]. Importantly, higher adjusted serum calcium concentrations were also associated with an increased risk of death, independent of serum phosphorus. In the same study, only moderate to severe hyperparathyroidism (PTH concentrations ≥600 pg/mL), but not more modest increases in PTH, was associated with an increase in the relative risk of death. Hyperphosphatemia and hyperparathyroidism were significantly associated with all-cause, cardiovascular, and fracture-related hospitalization in dialysis patients.

A recent post-hoc data analysis from the Multiple Outcomes of Raloxifene Evaluation (MORE) trial of raloxifene treatment in 7259 postmenopausal women with osteoporosis examined the associations between higher baseline calcium and phosphorus levels and incident cardiovascular events over 4 years [69]. After adjustment for multiple covariates, including 25(OH)D, parathyroid hormone, and phosphorus, adjusted hazard ratios (95% confidence interval) per SD of calcium were significantly increased for combined cardiovascular outcomes [AHR 1.17 (95% CI, 1.01-1.35), p = 0.03], but were either marginal for cerebrovascular events [1.22 (95% CI, 0.99-1.49), p = 0.06], or not significant for coronary heart disease [1.12 (95% CI, 0.92-1.37), p = 0.25], and death [1.18 (95% CI, 0.94-1.48), p = 0.16]. Associations between serum phosphorus and cardiovascular events did not persist after adjustment for additional confounders. Thus, there was an independent association between higher serum calcium levels, but not higher serum phosphorus levels, and higher rates of cardiovascular events in postmenopausal women with osteoporosis. It is interesting to speculate that greater increases in serum calcium resulting from calcium citrate than calcium carbonate may be responsible for the findings in the Auckland study of postmenopausal women.

## 8. Conclusion

In conclusion, the findings from this review that evaluated the results from observational and prospective studies, RCTs and meta-analyses, highlight the heterogeneity of findings regarding whether the use of calcium supplements has any adverse effects of health, including kidney stones, vascular disease and mortality. In terms of CVD and its related risk factors, the vast majority of studies have failed to observe any significant adverse effect of calcium supplement use. However, the findings from a recent meta-analysis of published RCTs (currently published in abstract form only) that included most of the key calcium supplementation trials having cardiovascular events as secondary outcomes, provide the most compelling data to support the notion that the use of calcium supplements may increase the risk of MI in postmenopausal women [22]. While these findings are of concern, the clinical and public health implications need to be carefully considered. What is the key message for physicians, health care professionals and patients? We believe that it would be unwise at this point to recommend that older women avoid or stop taking their calcium supplements. Unfortunately, this may well be the key message conveyed when the findings from this meta-analysis are published. 

When evaluating whether calcium supplements adversely affect vascular disease, it is important to consider that total calcium intakes may exceed 2,000 to 2,500 mg per day (1,000–1,200 mg/d supplemental calcium plus 800–1,000 mg/d dietary calcium). At these levels, there may well be some cause for concern, but several important questions still remain. Is the level of risk the same for all women or is it greatest in those already at high risk of CVD? Does the level of risk vary by the type of calcium supplement used, particularly those that led to greater elevations of serum calcium concentrations, and does the addition of vitamin D counteract this increased risk? Is there a threshold level of calcium above which intakes become detrimental to health? Although there is currently no evidence to support such a threshold, a recent study in healthy adult men and women aged 19 to 75 years which was designed to determine the level of dietary calcium to maintain neutral calcium balance, reported that the calcium requirements (or recommended dietary allowance) for men and women should be approximately 1035 mg/d [8]. While some have questioned the balance-based approach used to estimate the average calcium requirements in this study [70], these findings provide some evidence that daily calcium requirements may be lower than previously estimated. 

It is evident from the above discussion that there are still many important unanswered questions that need to be addressed before women are advised to stop taking their calcium supplements. Unfortunately it is unlikely that there will ever be a long-term RCTs conducted to address whether the use of calcium supplements is associated with increased vascular disease because the primary hypothesis would be one of harm  [31]. Therefore, large cohort prospective studies comparing groups with habitually high *versus* low calcium intakes (or those regularly taking calcium supplements) will be important to gain an insight into the long-term effects of high calcium intakes on vascular disease and related mortality. Until then, we recommend that healthy older women adhere to the current guidelines which typically recommend a total calcium intake of 1,000 to 1,300 mg/d, which can be readily achieved through both dietary sources and the addition of calcium supplements, when required. 

## References

[1] Daly R.M., Bass S., Nowson C. (2006). Long-term effects of calcium-vitamin-D3-fortified milk on bone geometry and strength in older men. Bone.

[2] Daly R.M., Brown M., Bass S., Kukuljan S., Nowson C. (2006). Calcium- and vitamin D3-fortified milk reduces bone loss at clinically relevant skeletal sites in older men: a 2-year randomized controlled trial. J. Bone Miner. Res..

[3] Prince R.L., Devine A., Dhaliwal S.S., Dick I.M. (2006). Effects of calcium supplementation on clinical fracture and bone structure: results of a 5-year, double-blind, placebo-controlled trial in elderly women. Arch. Intern. Med..

[4] Reid I.R., Ames R., Mason B., Reid H.E., Bacon C.J., Bolland M.J., Gamble G.D., Grey A., Horne A. (2008). Randomized controlled trial of calcium supplementation in healthy, nonosteoporotic, older men. Arch. Intern. Med..

[5] Riggs B.L., O'Fallon W.M., Muhs J., O'Connor M.K., Kumar R., Melton L.J. (1998). Long-term effects of calcium supplementation on serum parathyroid hormone level, bone turnover, and bone loss in elderly women. J. Bone Miner. Res..

[6] Tang B.M., Eslick G.D., Nowson C., Smith C., Bensoussan A. (2007). Use of calcium or calcium in combination with vitamin D supplementation to prevent fractures and bone loss in people aged 50 years and older: a meta-analysis. Lancet.

[7] Food and Nutrition Board, Institute of Medicine (1997). Dietary Reference Intakes for Calcium, Phosphorus, Magnesium, Vitamin D, and Fluoride.

[8] Hunt C.D., Johnson L.K. (2007). Calcium requirements: new estimations for men and women by cross-sectional statistical analyses of calcium balance data from metabolic studies. Am. J. Clin. Nutr..

[9] Jackson R.D., LaCroix A.Z., Gass M., Wallace R.B., Robbins J., Lewis C.E., Bassford T., Beresford S.A., Black H.R., Blanchette P., Bonds D.E., Brunner R.L., Brzyski R.G., Caan B., Cauley J.A., Chlebowski R.T., Cummings S.R., Granek I., Hays J., Heiss G., Hendrix S.L., Howard B.V., Hsia J., Hubbell F.A., Johnson K.C., Judd H., Kotchen J.M., Kuller L.H., Langer R.D., Lasser N.L., Limacher M.C., Ludlam S., Manson J.E., Margolis K.L., McGowan J., Ockene J.K., O'Sullivan M.J., Phillips L., Prentice R.L., Sarto G.E., Stefanick M.L., Van Horn L., Wactawski-Wende J., Whitlock E., Anderson G.L., Assaf A.R., Barad D. (2006). Calcium plus vitamin D supplementation and the risk of fractures. N. Engl. J. Med..

[10] LaCroix A.Z., Kotchen J., Anderson G., Brzyski R., Cauley J.A., Cummings S.R., Gass M., Johnson K.C., Ko M., Larson J., Manson J.E., Stefanick M.L., Wactawski-Wende J. (2009). Calcium plus vitamin D supplementation and mortality in postmenopausal women: the Women's Health Initiative calcium-vitamin D randomized controlled trial. J. Gerontol. A. Biol. Sci. Med. Sci..

[11] Pittas A.G., Lau J., Hu F.B., Dawson-Hughes B. (2007). The role of vitamin D and calcium in type 2 diabetes. A systematic review and meta-analysis. J. Clin. Endocrinol. Metab..

[12] Lappe J.M., Travers-Gustafson D., Davies K.M., Recker R.R., Heaney R.P. (2007). Vitamin D and calcium supplementation reduces cancer risk: results of a randomized trial. Am. J. Clin. Nutr..

[13] Baron J.A., Beach M., Mandel J.S., van Stolk R.U., Haile R.W., Sandler R.S., Rothstein R., Summers R.W., Snover D.C., Beck G.J., Bond J.H., Greenberg E.R. (1999). Calcium supplements for the prevention of colorectal adenomas. Calcium Polyp Prevention Study Group. N. Engl. J. Med..

[14] Bostick R.M., Kushi L.H., Wu Y., Meyer K.A., Sellers T.A., Folsom A.R. (1999). Relation of calcium, vitamin D, and dairy food intake to ischemic heart disease mortality among postmenopausal women. Am. J. Epidemiol..

[15] Bell L., Halstenson C.E., Halstenson C.J., Macres M., Keane W.F. (1992). Cholesterol-lowering effects of calcium carbonate in patients with mild to moderate hypercholesterolemia. Arch. Intern. Med..

[16] Denke M.A., Fox M.M., Schulte M.C. (1993). Short-term dietary calcium fortification increases fecal saturated fat content and reduces serum lipids in men. J. Nutr..

[17] Reid I.R., Mason B., Horne A., Ames R., Clearwater J., Bava U., Orr-Walker B., Wu F., Evans M.C., Gamble G.D. (2002). Effects of calcium supplementation on serum lipid concentrations in normal older women: a randomized controlled trial. Am. J. Med..

[18] Griffith L.E., Guyatt G.H., Cook R.J., Bucher H.C., Cook D.J. (1999). The influence of dietary and nondietary calcium supplementation on blood pressure: an updated metaanalysis of randomized controlled trials. Am. J. Hypertens..

[19] van Mierlo L.A., Arends L.R., Streppel M.T., Zeegers M.P., Kok F.J., Grobbee D.E., Geleijnse J.M. (2006). Blood pressure response to calcium supplementation: a meta-analysis of randomized controlled trials. J. Hum. Hypertens..

[20] Bolland M.J., Barber P.A., Doughty R.N., Mason B., Horne A., Ames R., Gamble G.D., Grey A., Reid I.R. (2008). Vascular events in healthy older women receiving calcium supplementation: randomised controlled trial. BMJ.

[21] Pentti K., Tuppurainen M.T., Honkanen R., Sandini L., Kroger H., Alhava E., Saarikoski S. (2009). Use of calcium supplements and the risk of coronary heart disease in 52-62-year-old women: The Kuopio Osteoporosis Risk Factor and Prevention Study. Maturitas.

[22] Reid I.R., Bolland M.J., Grey A. (2010). The calcium controversy: balancing heart and bone effects of supplements. Int. Med. J..

[23] German J.B., Gibson R.A., Krauss R.M., Nestel P., Lamarche B., van Staveren W.A., Steijns J.M., de Groot L.C., Lock A.L., Destaillats F. (2009). A reappraisal of the impact of dairy foods and milk fat on cardiovascular disease risk. Eur. J. Nutr..

[24] Gibson R.A., Makrides M., Smithers L.G., Voevodin M., Sinclair A.J. (2009). The effect of dairy foods on CHD: a systematic review of prospective cohort studies. Br. J. Nutr..

[25] Al-Delaimy W.K., Rimm E., Willett W.C., Stampfer M.J., Hu F.B. (2003). A prospective study of calcium intake from diet and supplements and risk of ischemic heart disease among men. Am. J. Clin. Nutr..

[26] Ascherio A., Rimm E.B., Hernan M.A., Giovannucci E.L., Kawachi I., Stampfer M.J., Willett W.C. (1998). Intake of potassium, magnesium, calcium, and fiber and risk of stroke among US men. Circulation.

[27] Iso H., Stampfer M.J., Manson J.E., Rexrode K., Hennekens C.H., Colditz G.A., Speizer F.E., Willett W.C. (1999). Prospective study of calcium, potassium, and magnesium intake and risk of stroke in women. Stroke.

[28] Nurmi-Luthje I., Luthje P., Kaukonen J.P., Kataja M., Kuurne S., Naboulsi H., Karjalainen K. (2009). Post-fracture prescribed calcium and vitamin D supplements alone or, in females, with concomitant anti-osteoporotic drugs is associated with lower mortality in elderly hip fracture patients: a prospective analysis. Drugs Aging.

[29] Jones G., Winzenberg T. (2008). Cardiovascular risks of calcium supplements in women. BMJ.

[30] Bolland M.J., Grey A.B., Reid I.R. (2008). Re: Calcium supplementation does not increase mortality. Med. J. Aust..

[31] Reid I.R., Bolland M.J., Grey A. (2010). Does Calcium Supplementation Increase Cardiovascular Risk?. Clin. Endocrinol. (Oxf)..

[32] Andrews N.A. (2008). Calcium supplementation and vascular disease: A legitimate new worry?. IBMS BoneKEy..

[33] Sabbagh Z., Vatanparast H. (2009). Is calcium supplementation a risk factor for cardiovascular diseases in older women?. Nutr. Rev..

[34] Lappe J.M., Heaney R.P. (2008). Calcium supplementation: Results may not be generalisable. BMJ.

[35] Hanzlik R.P., Fowler S.C., Fisher D.H. (2005). Relative bioavailability of calcium from calcium formate, calcium citrate, and calcium carbonate. J. Pharmacol. Exp. Ther..

[36] Reid I.R., Schooler B.A., Hannan S.F., Ibbertson H.K. (1986). The acute biochemical effects of four proprietary calcium preparations. Aust. N. Z. J. Med..

[37] Ginde A.A., Scragg R., Schwartz R.S., Camargo C.A. (2009). Prospective study of serum 25-hydroxyvitamin D level, cardiovascular disease mortality, and all-cause mortality in older U.S. adults. J. Am. Geriatr. Soc..

[38] Kilkkinen A., Knekt P., Aro A., Rissanen H., Marniemi J., Heliovaara M., Impivaara O., Reunanen A. (2009). Vitamin D status and the risk of cardiovascular disease death. Am. J. Epidemiol..

[39] Grant A.M., Avenell A., Campbell M.K., McDonald A.M., MacLennan G.S., McPherson G.C., Anderson F.H., Cooper C., Francis R.M., Donaldson C., Gillespie W.J., Robinson C.M., Torgerson D.J., Wallace W.A. (2005). Oral vitamin D3 and calcium for secondary prevention of low-trauma fractures in elderly people (Randomised Evaluation of Calcium Or vitamin D, RECORD): a randomised placebo-controlled trial. Lancet.

[40] Brazier M., Grados F., Kamel S., Mathieu M., Morel A., Maamer M., Sebert J.L., Fardellone P. (2005). Clinical and laboratory safety of one year's use of a combination calcium + vitamin D tablet in ambulatory elderly women with vitamin D insufficiency: results of a multicenter, randomized, double-blind, placebo-controlled study. Clin. Ther..

[41] Hsia J., Heiss G., Ren H., Allison M., Dolan N.C., Greenland P., Heckbert S.R., Johnson K.C., Manson J.E., Sidney S., Trevisan M. (2007). Calcium/vitamin D supplementation and cardiovascular events. Circulation.

[42] Zhu K., Bruce D., Austin N., Devine A., Ebeling P.R., Prince R.L. (2008). Randomized controlled trial of the effects of calcium with or without vitamin D on bone structure and bone-related chemistry in elderly women with vitamin D insufficiency. J. Bone Miner. Res..

[43] Autier P., Gandini S. (2007). Vitamin D supplementation and total mortality: a meta-analysis of randomized controlled trials. Arch. Intern. Med..

[44] Wang L., Manson J.E., Song Y., Sesso H.D. (2010). Systematic review: Vitamin D and calcium supplementation in prevention of cardiovascular events. Ann. Intern. Med..

[45] Govers M.J., Van der Meet R. (1993). Effects of dietary calcium and phosphate on the intestinal interactions between calcium, phosphate, fatty acids, and bile acids. Gut.

[46] Zemel M.B., Shi H., Greer B., Dirienzo D., Zemel P.C. (2000). Regulation of adiposity by dietary calcium. FASEB J..

[47] Zemel M.B. (2001). Calcium modulation of hypertension and obesity: mechanisms and implications. J. Am. Coll. Nutr..

[48] Reid I.R., Horne A., Mason B., Ames R., Bava U., Gamble G.D. (2005). Effects of calcium supplementation on body weight and blood pressure in normal older women: a randomized controlled trial. J. Clin. Endocrinol. Metab..

[49] Allender P.S., Cutler J.A., Follmann D., Cappuccio F.P., Pryer J., Elliott P. (1996). Dietary calcium and blood pressure: a meta-analysis of randomized clinical trials. Ann. Intern. Med..

[50] Wilson P.W., Kauppila L.I., O'Donnell C.J., Kiel D.P., Hannan M., Polak J.M., Cupples L.A. (2001). Abdominal aortic calcific deposits are an important predictor of vascular morbidity and mortality. Circulation.

[51] West S.L., Swan V.J., Jamal S.A. (2010). Effects of calcium on cardiovascular events in patients with kidney disease and in a healthy population. Clin. J. Am. Soc. Nephrol..

[52] Chertow G.M., Burke S.K., Raggi P. (2002). Sevelamer attenuates the progression of coronary and aortic calcification in hemodialysis patients. Kidney Int..

[53] Russo D., Miranda I., Ruocco C., Battaglia Y., Buonanno E., Manzi S., Russo L., Scafarto A., Andreucci V.E. (2007). The progression of coronary artery calcification in predialysis patients on calcium carbonate or sevelamer. Kidney Int..

[54] Bhakta M., Bruce C., Messika-Zeitoun D., Bielak L., Sheedy P.F., Peyser P., Sarano M. (2009). Oral calcium supplements do not affect the progression of aortic valve calcification or coronary artery calcification. J. Am. Board Fam. Med..

[55] Van Pelt N., Ruygrok P., Bolland M.J., Gamble G.D., Mason B., Ames R., Reid I.R. (2009). Do calcium supplements lead to an increase in coronary calcification?. Heart, Lung Circulation.

[56] Daly R.M., R. E.P., Khan B., Nowson C.A. (2009). Effects of calcium-vitamin D3 fortified milk on abdominal aortic calcification in older men: Retrospective analysis of a 2-year randomised controlled trial. J. Bone Miner. Res..

[57] Kiechl S., Schett G., Wenning G., Redlich K., Oberhollenzer M., Mayr A., Santer P., Smolen J., Poewe W., Willeit J. (2004). Osteoprotegerin is a risk factor for progressive atherosclerosis and cardiovascular disease. Circulation.

[58] Nitta K., Akiba T., Uchida K., Otsubo S., Takei T., Yumura W., Kabaya T., Nihei H. (2004). Serum osteoprotegerin levels and the extent of vascular calcification in haemodialysis patients. Nephrol. Dial. Transplant..

[59] Clancy P., Oliver L., Jayalath R., Buttner P., Golledge J. (2006). Assessment of a serum assay for quantification of abdominal aortic calcification. Arterioscler. Thromb. Vasc. Biol..

[60] Marque S., Jacqmin-Gadda H., Dartigues J.F., Commenges D. (2003). Cardiovascular mortality and calcium and magnesium in drinking water: an ecological study in elderly people. Eur. J. Epidemiol..

[61] Payne M.E., Anderson J.J., Steffens D.C. (2008). Calcium and vitamin D intakes may be positively associated with brain lesions in depressed and nondepressed elders. Nutr Res..

[62] Payne M.E., Haines P.S., Chambless L.E., Anderson J.J., Steffens D.C. (2007). Food group intake and brain lesions in late-life vascular depression. Int. Psychogeriatr..

[63] Curhan G.C., Willett W.C., Rimm E.B., Stampfer M.J. (1993). A prospective study of dietary calcium and other nutrients and the risk of symptomatic kidney stones. N. Engl. J. Med..

[64] Siener R., Glatz S., Nicolay C., Hesse A. (2003). Prospective study on the efficacy of a selective treatment and risk factors for relapse in recurrent calcium oxalate stone patients. Eur. Urol..

[65] Heaney R.P. (2008). Calcium supplementation and incident kidney stone risk: a systematic review. J. Am. Coll. Nutr..

[66] Lind L., Skarfors E., Berglund L., Lithell H., Ljunghall S. (1997). Serum calcium: a new, independent, prospective risk factor for myocardial infarction in middle-aged men followed for 18 years. J. Clin. Epidemiol..

[67] Foley R.N., Collins A.J., Ishani A., Kalra P.A. (2008). Calcium-phosphate levels and cardiovascular disease in community-dwelling adults: the Atherosclerosis Risk in Communities (ARIC) Study. Am. Heart J..

[68] Block G.A., Klassen P.S., Lazarus J.M., Ofsthun N., Lowrie E.G., Chertow G.M. (2004). Mineral metabolism, mortality, and morbidity in maintenance hemodialysis. J. Am. Soc. Nephrol..

[69] Slinin Y., Blackwell T., Ishani A., Cummings S.R., Ensrud K.E. (2010). Serum calcium, phosphorus and cardiovascular events in post-menopausal women. Int. J. Cardiol..

[70] Heaney R.P. (2007). Mineral balance and mineral requirement. Am. J. Clin. Nutr..

